# Role of glycolysis related genes in the pathogenesis of hemorrhoids and immune cell infiltration analysis

**DOI:** 10.1038/s41598-025-18382-3

**Published:** 2025-09-25

**Authors:** Peng Li, Qian Hou, Xiaodong Yang, Wenbin Han, Hao Wang

**Affiliations:** 1 Department of Colorectal and General Surgery, Qujiang Branch, Xi’an Hospital of Traditional Chinese Medicine, Xi’an, 710061 China; 2 Department of General Practice, Xi’an People’s Hospital (Xi’an Fourth Hospital), Xi’an, 710004 China

**Keywords:** Hemorrhoids, Glycolysis, Differentially expressed genes, Immune infiltration, Biological functions, Gastrointestinal diseases, Inflammation, Molecular biology, Glycobiology

## Abstract

Hemorrhoids are a prevalent condition affecting the anorectal area. Recent studies have highlighted glycolysis as a crucial metabolic pathway in numerous diseases. However, systematic studies exploring the distinct functions of the seven glycolysis-related genes (GRGs) during hemorrhoid development are limited. This investigation sought to elucidate the function of GRGs in hemorrhoid development and their correlation with immune cell infiltration. Using bioinformatics methodologies, we performed differential expression analysis, Gene Ontology (GO) and Kyoto Encyclopedia of Genes and Genomes (KEGG) enrichment analyses, gene set enrichment analysis (GSEA), establishment of protein–protein interaction (PPI) networks, and analysis of immune infiltration. We identified 34 glycolysis-related differentially expressed genes (GRDEGs) in the GSE154650 dataset, including *PCK1*, *ALDOB*, and *PCK2*. GO and KEGG analyses showed a considerable increase of GRDEGs in monosaccharide and glucose metabolic processes and AMPK signaling cascades. PPI network analysis identified seven hub genes (HGs) that may act as essential regulatory nodes and potential drug targets. Additionally, we found notable associations between the infiltration patterns of monocytes and plasma cells and particular HGs, highlighting the significance of the immune microenvironment. This study established a foundation for subsequent functional validation and exploration of innovative therapeutic strategies targeting glycolysis-related pathways in hemorrhoids.

**Supplementary Information:**

The online version contains supplementary material available at 10.1038/s41598-025-18382-3.

## Introduction

Hemorrhoids are one of the most frequently encountered disorders in the field of proctology, with a prevalence varying between 4.4 and 40%. Clinical symptoms associated with this condition include bleeding, prolapse, edema, and discomfort. These manifestations notably diminish the well-being of affected individuals and also result in substantial financial burden^1,2^. Existing therapeutic approaches encompass a combination of medical and surgical treatments; however, their effectiveness is frequently hindered by high recurrence rates and varying degrees of patient satisfaction. There is no universally accepted treatment consensus for hemorrhoids; therefore, management is individualized globally^3^. Consequently, investigating novel molecular pathways and promising therapeutic interventions is essential for enhancing clinical outcomes.

Glycolysis is the most important pathway of cellular metabolism for energy production and biosynthesis. Previous research has indicated that glycolysis is not only crucial for the energy metabolism of healthy cells but is also significantly connected to the development and advancement of multiple disorders, encompassing cancer, metabolic disorders, and inflammatory conditions^4^. In immune cells, enhanced glycolysis is crucial for their function, especially in response to infections and tumors, and metabolic reprogramming of immune cells is often accompanied by increased glycolysis^5^. Although significant attention has been devoted to understanding the implications of glycolytic changes in malignancies and metabolic syndromes, the precise relationship between glycolytic gene expression and hemorrhoidal disease remains inadequately characterized. This lack of knowledge underscores the need for further exploration of metabolic abnormalities associated with hemorrhoids and their possible contributions to disease mechanisms.

This study used a bioinformatics approach, including differential expression, Gene Ontology (GO) and Kyoto Encyclopedia of Genes and Genomes (KEGG) enrichment analyses, gene set enrichment analysis (GSEA), development of protein–protein interaction (PPI) networks, and immune infiltration evaluation, to explore glycolysis-related genes (GRGs) and hemorrhoids. We sought to detect critical metabolic pathways and genes that could serve as potential biomarkers and treatment targets, while also deepening our understanding of the role of glycolytic metabolism in hemorrhoids.

## Results

### Technology roadmap

**Fig. 1:**
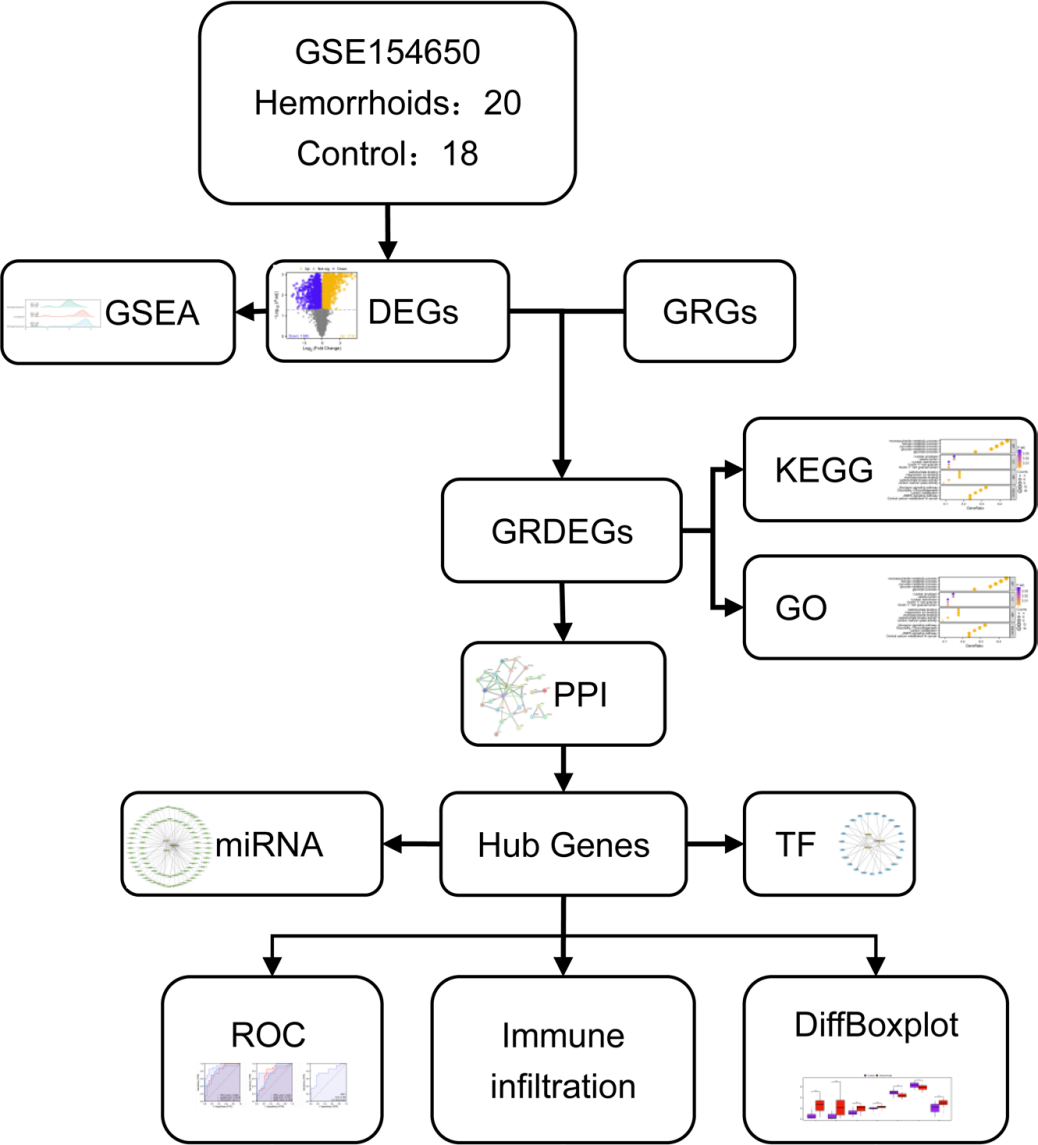
Flowchart. GSEA, Gene Set Enrichment Analysis; DEGs, Differentially Expressed Genes; GRGs, Glycolysis-Related Genes; KEGG, Kyoto Encyclopedia of Genes and Genomes; GO, Gene Ontology; GRDEGs, Glycolysis-Related Differentially Expressed Genes; PPI, Protein–protein Interaction; TF, Transcription Factor; ROC, Receiver Operating Characteristic; DiffBoxplot, Differentially Boxplot.

The study workflow is presented in Fig. 1.

### Glycolysis-related differentially expressed genes (GRDEGs)

**Fig. 2:**
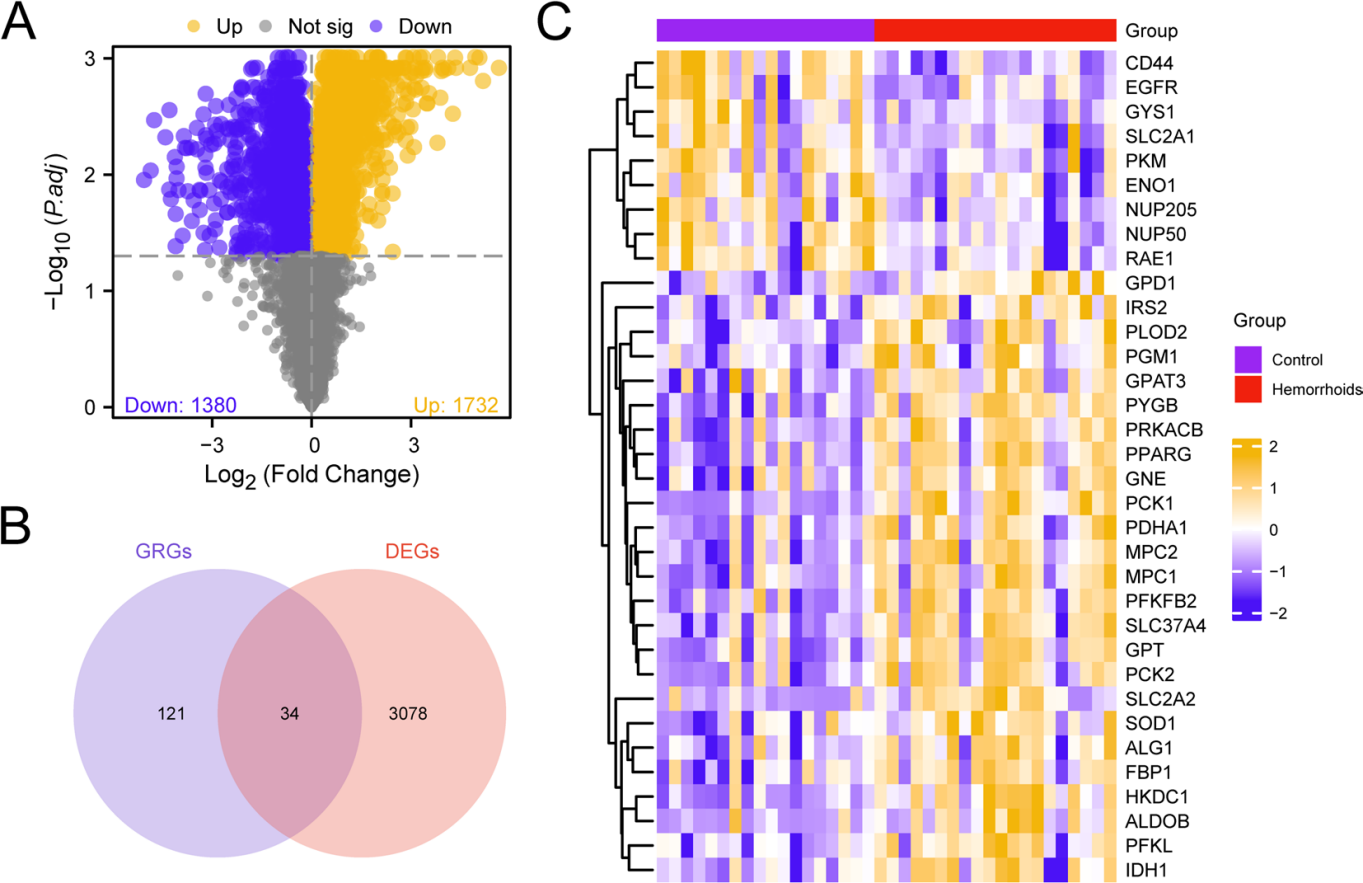
Differential Gene expression analysis. (**A**) Differential gene expression analysis Volcano map of hemorrhoids and anal fissure (Control) samples in dataset GSE154650. (**B**) Venn diagram of DEGs and GRGs in dataset GSE154650. (**C**) Heat map of GRDEGs in the dataset GSE154650. DEGs, Differentially Expressed Genes; GRGs, Glycolysis-Related Genes; GRDEGs, Glycolysis-Related Differentially Expressed Genes. Purple is the anal fissure (Control) sample, red is the hemorrhoids sample. The heat map shows orange and yellow for high expression and blue for low expression.

The dataset GSE154650 was divided into hemorrhoids and anal fissure (control) samples (Table 1). To investigate the gene expression, the DEGs of hemorrhoids and control samples in the dataset GSE154650 were obtained by using R package limma for differential analysis, and the outcomes were: A total of 3112 DEGs were acquired in the GSE154650 dataset, fulfilling the criteria of |logFC| > 0 and adjusted *p*-value < 0.05. Within the analyzed genes, 1732 displayed upregulation (logFC > 0 and adj.*p* < 0.05), while 1380 demonstrated downregulation (logFC < 0 and adj.*p* < 0.05). A volcano plot was developed per the differential analysis outcomes derived from this dataset (Fig. 2A).

**Table 1:** GEO microarray chip Information.

	GSE154650
Platform	GPL20301
Species	Homo sapiens
Tissue	Hemorrhoid Tissue
Samples in Hemorrhoids group	20
Samples in Control group	18
Reference	PMID: 3,388,516

GEO, Gene Expression Omnibus.

To obtain GRDEGs, all the obtained |logFC| >0 and adj. *p* < 0.05 DEGs and GRGs were plotted and a Venn diagram (Fig. 2B) was generated. A total of 34 GRDEGs were identified: *PCK1*, *PYGB*, *HKDC1*, *GPT*, *ALDOB*, *CD44*, *PRKACB*, *PPARG*, *SLC37A4*, *MPC2*, *PCK2*, *PLOD2*, *IRS2*, *MPC1*, *PFKFB2*, *PFKL*, *EGFR*, *GPD1*, *NUP205*, *PKM*, *SOD1*, *GYS1*, *GNE*, *NUP50*, *SLC2A1*, *PDHA1*, *RAE1*, *IDH1*, *ALG1*, *SLC2A2*, *GPAT3*, *ENO1*, *PGM1*, and *FBP1*. Based on the findings of the intersection analysis, the expression patterns of GRDEGs among diverse sample categories in the GSE154650 dataset were investigated. The analysis outcomes are illustrated in heat maps created utilizing the pheatmap package for R (Fig. 2C).

### GO and KEGG pathway enrichment analysis

**Fig. 3:**
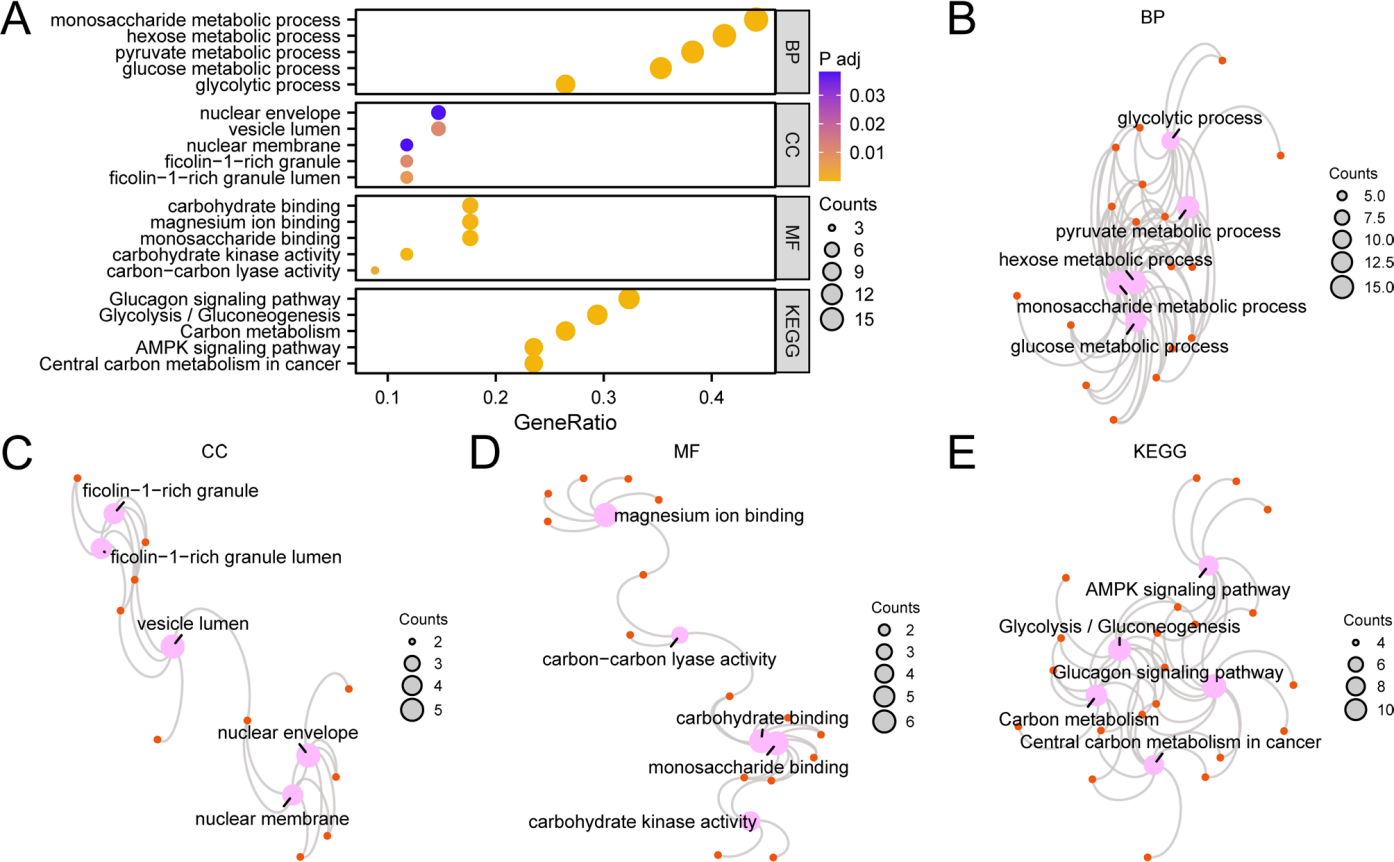
(**A**) GO and KEGG Enrichment Analysis for GRDEGs. The bubble chart displays GO and KEGG enrichment analysis findings of GRDEGs, depicting BP, CC, MF, and KEGG categories. The y-axis indicates various GO and KEGG classifications. (**B**–**E**). Network diagrams illustrate GO and KEGG enrichment analysis outcomes for GRDEGs: BP (**B**), CC (**C**), MF (**D**), and KEGG (**E**). Pink elements denote entries, orange components indicate molecules, while connecting lines demonstrate entry-molecule associations. GRDEGs, Glycolysis-Related Differentially Expressed Genes; GO, Gene Ontology; KEGG, Kyoto Encyclopedia of Genes and Genomes; BP, Biological Process; CC, Cellular Component; MF, Molecular Function. Within the bubble visualization, bubble dimensions correlate with gene quantities, while bubble coloration reflects adj. *p*-value magnitude. Yellower shades signify lower adj. *p*-values; bluer tones indicate higher adj. *p*-values. The selection parameters for gene body (GO) and pathway (KEGG) enrichment analyses specified adj. *p* < 0.05 and FDR value (q value) < 0.25.

The link between biological process (BP), cellular component (CC), molecular function (MF) and the KEGG pathways of 34 GRDEGs and hemorrhoids was further explored through GO and KEGG pathway enrichment analysis. The data is depicted in Table 2. The examination indicated that the 34 GRDEGs in hemorrhoids were mainly enriched in the monosaccharide metabolic process, hexose metabolic process, pyruvate metabolic process, glucose metabolic process, and glycolytic process (BP); Cell components such as nuclear envelope, vesicle lumen, nuclear membrane, ficolin − 1−rich granule, and ficolin − 1−rich lumen (CC); and carbohydrate binding, magnesium ion binding, monosaccharide binding, carbohydrate kinase activity, carbon − carbon lyase activity, and other MFs. It was also enriched in the glucagon signaling pathway, Glycolysis/Gluconeogenesis, Carbon metabolism, AMPK signaling cascade and Central carbon metabolism in cancer, and other biological pathways (KEGG). The outcomes of pathway enrichment investigation for GO terms and KEGG analyses are depicted through a bubble diagram (Fig. 3A).

**Table 2:** Results of GO and KEGG enrichment analysis for GRDEGs.

Ontology	ID	Description	GeneRatio	BgRatio	*p*-value	*p*.adjust
BP	GO:0006090	pyruvate metabolic process	13/34	106/18,800	2.29e−21	2.7e−18
BP	GO:0005996	monosaccharide metabolic process	15/34	261/18,800	1.34e−19	7.94e−17
BP	GO:0019318	hexose metabolic process	14/34	242/18,800	2.6e−18	1.03e−15
BP	GO:0006006	glucose metabolic process	12/34	201/18,800	7.15e−16	2.11e−13
BP	GO:0006096	glycolytic process	9/34	81/18,800	1.56e−14	3.44e−12
CC	GO:1,904,813	ficolin-1-rich granule lumen	4/34	124/19,594	6.12e−05	0.0070
CC	GO:0031983	vesicle lumen	5/34	327/19,594	0.0002	0.0110
CC	GO:0101002	ficolin-1-rich granule	4/34	185/19,594	0.0003	0.0110
CC	GO:0005635	nuclear envelope	5/34	479/19,594	0.0013	0.0381
CC	GO:0031965	nuclear membrane	4/34	300/19,594	0.0017	0.0382
MF	GO:0048029	monosaccharide binding	6/34	71/18,410	3.27e−09	4.95e−07
MF	GO:0019200	carbohydrate kinase activity	4/34	21/18,410	5.67e−08	4.28e−06
MF	GO:0000287	magnesium ion binding	6/34	222/18,410	2.91e−06	0.0001
MF	GO:0030246	carbohydrate binding	6/34	270/18,410	8.97e−06	0.0003
MF	GO:0016830	carbon-carbon lyase activity	3/34	50/18,410	0.0001	0.0030
KEGG	hsa00010	Glycolysis / Gluconeogenesis	10/34	67/8164	7.74e−14	1.17e−11
KEGG	hsa04922	Glucagon signaling pathway	11/34	107/8164	2.58e−13	1.95e−11
KEGG	hsa05230	Central carbon metabolism in cancer	8/34	70/8164	2.95e−10	1.48e−08
KEGG	hsa01200	Carbon metabolism	9/34	115/8164	6.22e−10	2.35e−08
KEGG	hsa04152	AMPK signaling pathway	8/34	121/8164	2.43e−08	7.33e−07

GO, Gene Ontology; BP, Biological Process; CC, Cellular Component; MF, Molecular Function; KEGG, Kyoto Encyclopedia of Genes and Genomes; GRDEGs, Glycolysis-Related Differentially Expressed Genes.

The network diagrams representing BP, CC, MF, and KEGG pathways emerged from GO and KEGG pathway enrichment analysis results (Fig. 3B–E). The connecting lines demonstrate related molecular components with annotations specific to each entry. Node dimensions reflect the quantity of molecules within each entry, with larger nodes signifying a greater number.

### GSEA

**Fig. 4:**
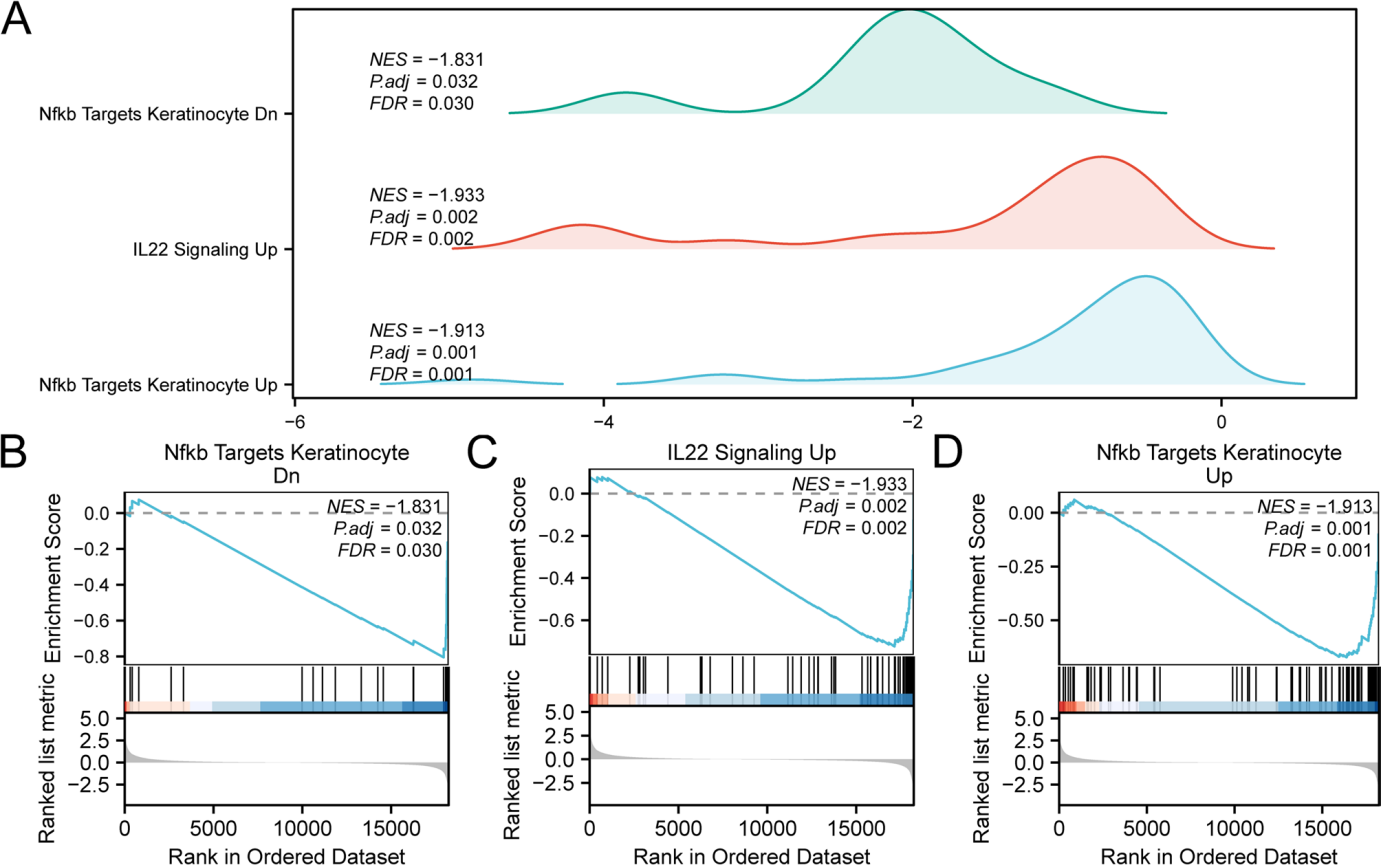
GSEA for GSE154650. (**A**) GSEA of the dataset GSE154650 shows three biological function mountain maps. (**B**–**D**) GSEA shows a significant enrichment of all genes in Nfkb Targets Keratinocyte Dn (**B**), IL22 Signaling Up (**C**), and Nfkb Targets Keratinocyte Up (**D**). GSEA, Gene Set Enrichment Analysis. The screening criteria for gene set enrichment analysis (GSEA) were adj.*p* < 0.05 and FDR (q value) < 0.25.

GSEA was applied to investigate the link between gene expression profiles in dataset GSE154650 and their corresponding BPs, CCs, and MFs (Fig. 4A). The comprehensive outcomes are depicted in Table 3. The findings suggested that every gene within the GSE154650 dataset exhibited markedly enrichment in Nfkb Targets Keratinocyte Dn (Fig. 4B), IL22 Signaling Up (Fig. 4C), Nfkb Targets Keratinocyte Up (Fig. 4D), along with multiple additional biologically relevant functions and signaling cascades.

**Table 3:** Results of GSEA for GSE154650.

ID	setSize	EnrichmentScore	NES	*p*-value	*p*.adjust	qvalue
REACTOME_KERATINIZATION	216	−0.8315390	−2.718981	1e−10	2.87e−08	2.64e−08
HOLLERN_SQUAMOUS_BREAST_TUMOR	173	−0.8387654	−2.645679	1e−10	2.87e−08	2.64e−08
JAEGER_METASTASIS_DN	251	−0.7965087	−2.623760	1e−10	2.87e−08	2.64e−08
BLANCO_MELO_BETA_INTERFERON_TREATED_BRONCHIAL_EPITHELIAL_CELLS_DN	186	−0.8106274	−2.586797	1e−10	2.87e−08	2.64e−08
REACTOME_FORMATION_OF_THE_CORNIFIED_ENVELOPE	128	−0.8489058	−2.577284	1e−10	2.87e−08	2.64e−08
VECCHI_GASTRIC_CANCER_ADVANCED_VS_EARLY_DN	136	0.8081626	2.410984	1e−10	2.87e−08	2.64e−08
HUPER_BREAST_BASAL_VS_LUMINAL_UP	53	−0.9032633	−2.377819	1e−10	2.87e−08	2.64e−08
SABATES_COLORECTAL_ADENOMA_DN	281	0.7354257	2.372178	1e−10	2.87e−08	2.64e−08
RICKMAN_TUMOR_DIFFERENTIATED_WELL_VS_POORLY_DN	365	−0.6928728	−2.345229	1e−10	2.87e−08	2.64e−08
REACTOME_BETA_DEFENSINS	38	−0.9011963	−2.243144	1e−10	2.87e−08	2.64e−08

GSEA, Gene Set Enrichment Analysis.

### Development of PPI networks and identification of hub genes (HGs)

**Fig. 5:**
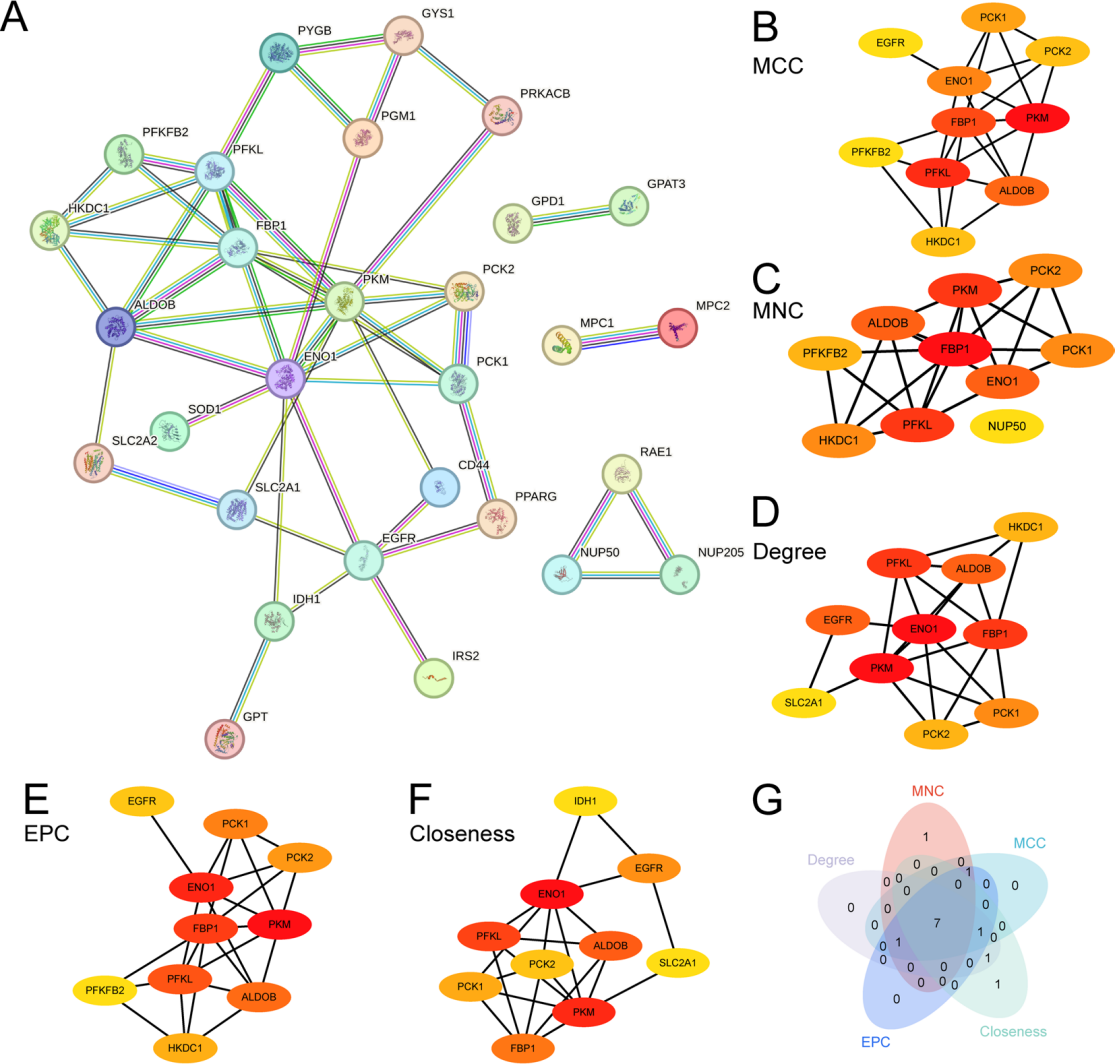
PPI Network and Hub Genes Analysis. (**A**) Protein–protein interaction (PPI) network of glycolysis-associated differentially expressed genes (GRDEGs) calculated using the STRING database. PPI network mapped by five algorithms (**B**–**F**). cytohubba plugin, including MCC (**B**), MNC (**C**), Degree (**D**), EPC (**E**), and Closeness (**F**). (**G**) cytohubba plugin’s Top GRDEGs Venn diagram of five algorithms.

First, an examination of the PPI was performed, resulting in the construction of a PPI network (Fig. 5A) comprising 34 GRDEGs. Analysis was performed utilizing the STRING database. The findings from the PPI Network indicated that 29 GRDEGs were related: *PYGB*, *GYS1*, *PGM1*, *PRKACB*, *GPD1*, *GPAT3*, *MPC1*, *MPC2*, *RAE1*, *NUP50*, *NUP205*, *HKCD1*, *ALDOB*, *PFKL*, *PKM*, *ENO1*, *PCK2*, *PCK1*, *PFKFB2*, *FBP1*, *SLC2A2*, *SOD1*, *SLC2A1*, *GPT*, *IDH1*, *EGFR*, *CD44*, *PPARG*, and *IRS2*. Subsequently, the scores of the 16 GRDEGs were calculated using five algorithms of the Cytoscape software’s cytohubba plugin, and the GRDEGs were ranked according to the scores. Next, the PPI network was mapped by applying the Top10 GRDEGs among the five algorithms, namely Maximal Clique Centrality (MCC) (Fig. 5B), Maximum Neighborhood Component (MNC) (Fig. 5C), Degree (Fig. 5D), Edge Percolated Component (EPC) (Fig. 5E), and Closeness (Fig. 5F). The gradient of the circle color, transitioning from red to yellow, illustrates the range of scores. Red indicates a high score and yellow indicates a low score. Finally, the genes identified by the five algorithms were compared, resulting in the creation of Fig. 5G. The intersecting genes from these algorithms represent HGs associated with the GRDEGs. The seven identified HGs were: *PCK1*, *ALDOB*, *PCK2*, *PFKL*, *PKM*, *ENO1*, and *FBP1*.

### Development of the regulatory network

**Fig. 6:**
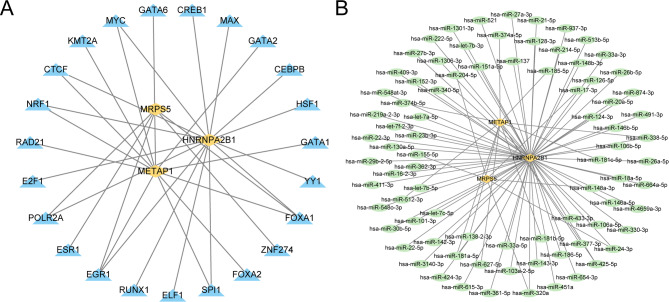
Regulatory Network of Hub Genes. (**A**) mRNA-TF Regulatory Network of hub genes. (**B**) mRNA-miRNA Regulatory Network of hub genes. TF: Transcription factor. Orange-yellow indicates mRNA, blue indicates TF, and green indicates miRNAs.

First, the transcription factors (TFs) linked to HGs were sourced from the ChIPBase database, and a regulatory network involving mRNA and TFs was constructed and illustrated utilizing the Cytoscape software (Fig. 6A). The network comprised three HGs and 23 TFs. Further comprehensive details are accessible in the online Supplementary Table S1 online.

Then, miRNAs associated with HGs were derived from the TarBase database, and a regulatory network involving mRNA and miRNAs was developed and visualized through Cytoscape software (Fig. 6B). We identified three HGs and 86 miRNAs. For more detailed information, refer to Supplementary Table S2 online.

**Fig. 7:**
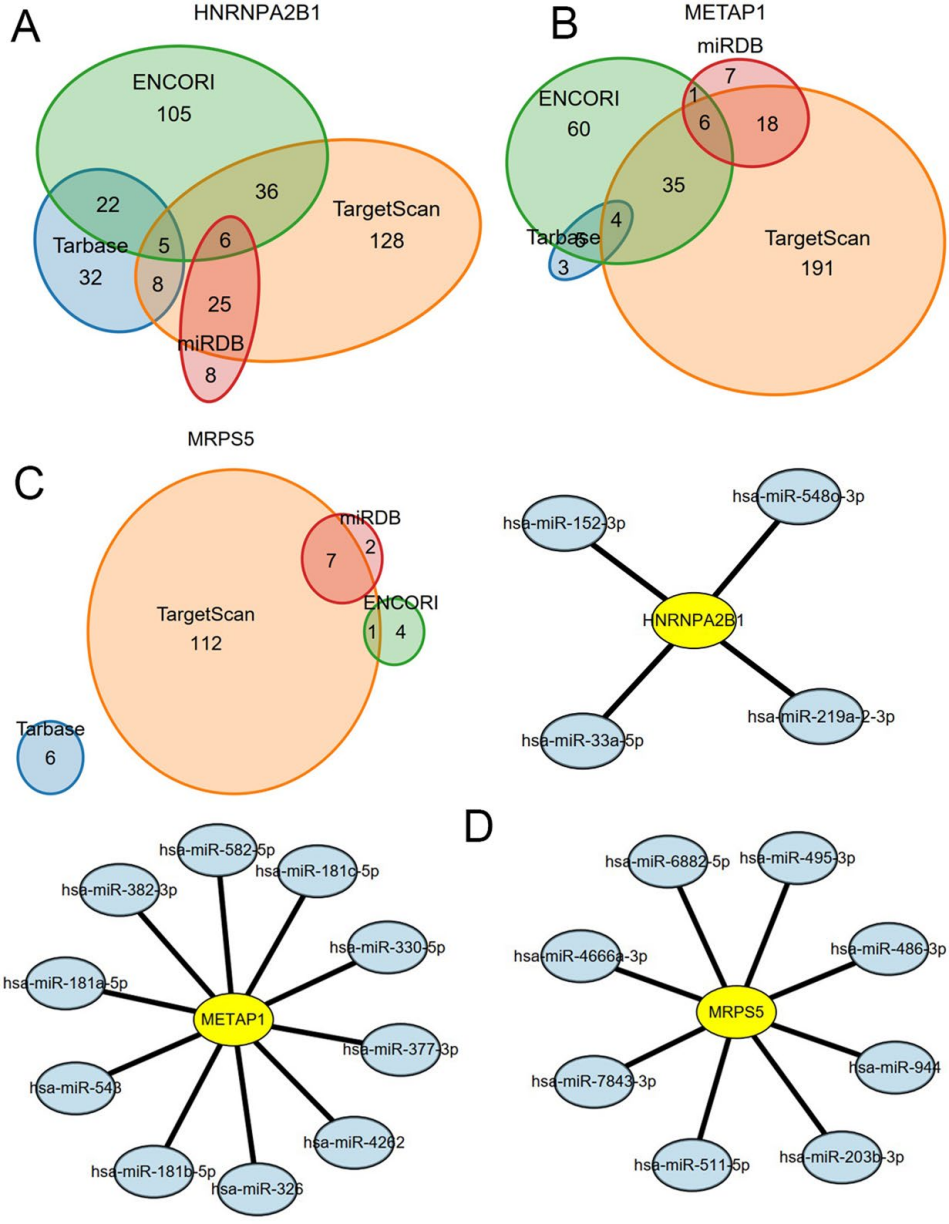
Multi-Database Integration Identifies Core miRNA Regulators of Glycolytic Hub Genes. Venn analysis of four databases (TargetScan, ENCORI, miRDB, and TarBase) identifies high-confidence upstream miRNAs targeting glycolytic hub genes: (**A**) 4 control HNRNPA2B1, (**B**) 10 miRNAs regulate METAP1, (**C**) 8 target MRPS5. The reconstructed mRNA-miRNA regulatory network (**D**) visualizes hub genes and their miRNA regulators. Circle areas in A-C scale proportionally to database-specific miRNA counts.

Then, to consolidate miRNA predictions, potential upstream miRNAs regulating HGs were retrieved from four databases: TargetScan, ENCORI, miRDB, and TarBase. Venn analysis identified intersection miRNAs across these databases, revealing 10 miRNAs for METAP1, 8 for MRPS5, and 4 for HNRNPA2B1 (Fig. 7A–C). Using this refined set, the mRNA-miRNA regulatory network (Fig. 7D) was reconstructed. Comprehensive miRNA identifiers are documented in Supplementary Table S3–S5 online.

### Verification of expression differences and receiver operating characteristic (ROC) curve assessment of HGs

**Fig. 8:**
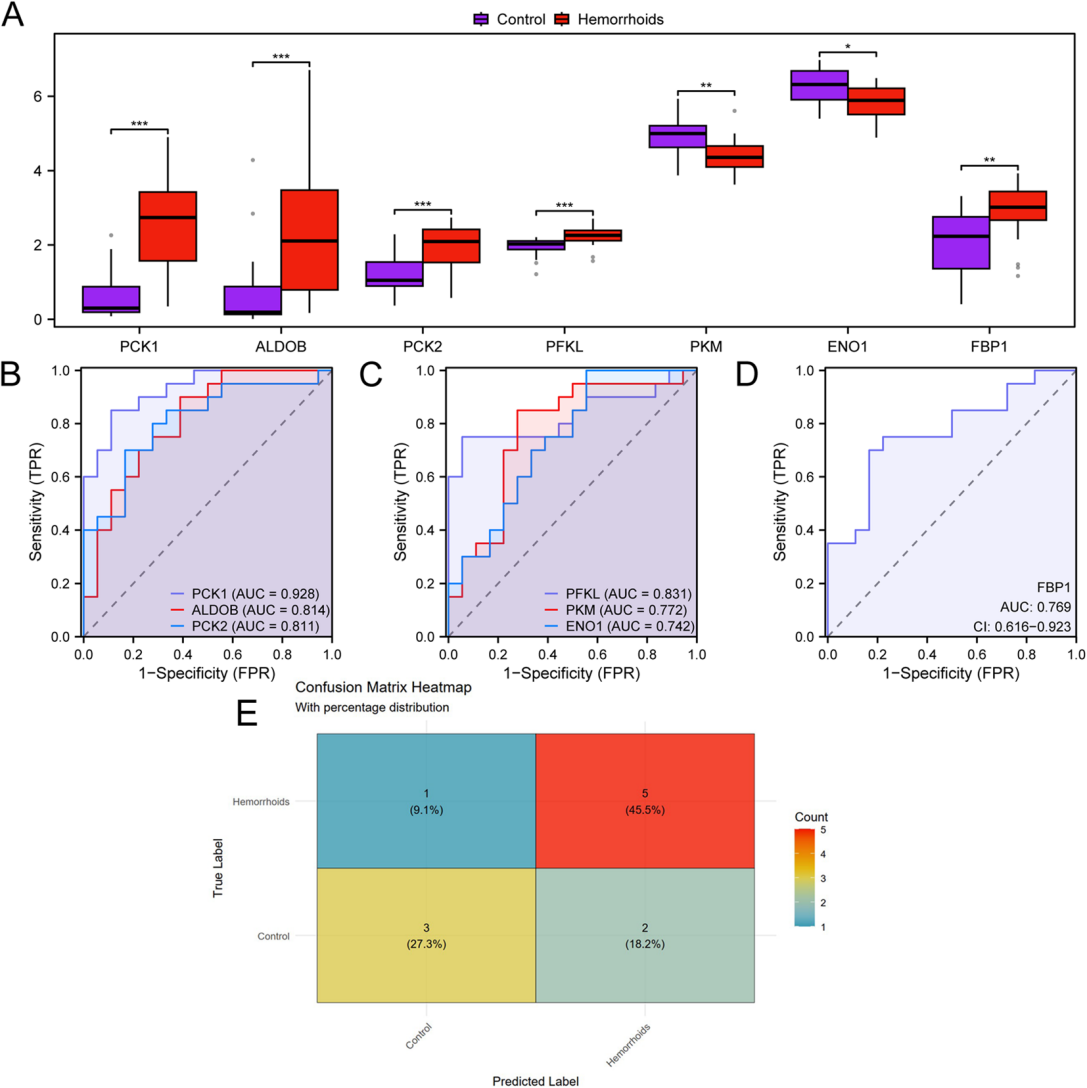
Differential Expression Validation, ROC Curve and Confusion Matrix Analysis. (**A**) Comparative clustering analysis of hub genes between hemorrhoids and control specimens in dataset GSE154650. (**B**–**D**) Receiver Operating Characteristic plots for hub genes *PCK1*, *ALDOB*, and *PCK2* (**B**), *PFKL*, *PKM*, and *ENO1* (**C**), *FBP1* (D) within dataset GSE154650. (**E**) Confusion matrix evaluating the diagnostic performance of the integrated 7-hub gene model, demonstrating a true positive rate of 45.5%, true negative rate of 27.3%, false positive rate of 18.2%, and false negative rate of 9.1%. * indicates *p*-value < 0.05, demonstrating statistical significance; ** indicates *p*-value < 0.01, showing strong statistical significance; *** indicates *p*-value < 0.001, revealing exceptional statistical significance. An AUC exceeding 0.5 suggests that molecular expression facilitates event occurrence. The proximity of AUC to 1 correlates with enhanced diagnostic capability. An AUC ranging from 0.7 to 0.9 signifies moderate diagnostic precision, while values surpassing 0.9 indicate superior diagnostic accuracy. GRDEGs, Glycolysis-Related Differentially Expressed Genes; ROC, Receiver Operating Characteristic; AUC, Area Under the Curve; TPR, True Positive Rate; FPR, False Positive Rate. Purple denotes anal fissures (Control) specimens and red signifies hemorrhoids specimens.

Figure 8A presents the expression patterns of seven HGs in samples derived from individuals with hemorrhoids and those with anal fissures (control) within the dataset GSE154650. The comparative investigation demonstrated that four HG levels (*PCK1*, *ALDOB*, *PCK2* and *PFKL*) in hemorrhoids and control samples in dataset GSE154650 were extremely statistically significant (*p*-value < 0.001), as presented in Fig. 8A. Two HGs levels, *PKM* and *FBP1*, demonstrated substantial statistical differences (*p*-value < 0.01) between hemorrhoid and control specimens within dataset GSE154650. Finally, one HG level, *ENO1*, in the hemorrhoids and control samples in dataset GSE154650 was statistically significant (*p*-value < 0.05). ROC curves were generated utilizing the R package pROC, which was informed by the expression levels of HGs within the dataset GSE154650. The ROC curve (Fig. 8B-D) manifested that HG *PCK1* level had high accuracy for the classification of hemorrhoids and control samples (area under the curve (AUC) > 0.9), and the expression levels of *ALDOB*, *PCK2*, *PFKL*, *PKM*, *ENO1*, and *FBP1* demonstrated a moderate level of accuracy (0.7 < AUC < 0.9) in distinguishing between hemorrhoids and anal fissures (controls) (AUC confidence interval in ROC analysis is provided as a Supplementary file). Confusion matrix analysis demonstrated the 7-HG integrated model performance: true positive rate 45.5%, true negative rate 27.3%, false positive rate 18.2%, and false negative rate 9.1% (Fig. 8E).

### Immunoinfiltration analysis of hemorrhoids (CIBERSORT)

**Fig. 9:**
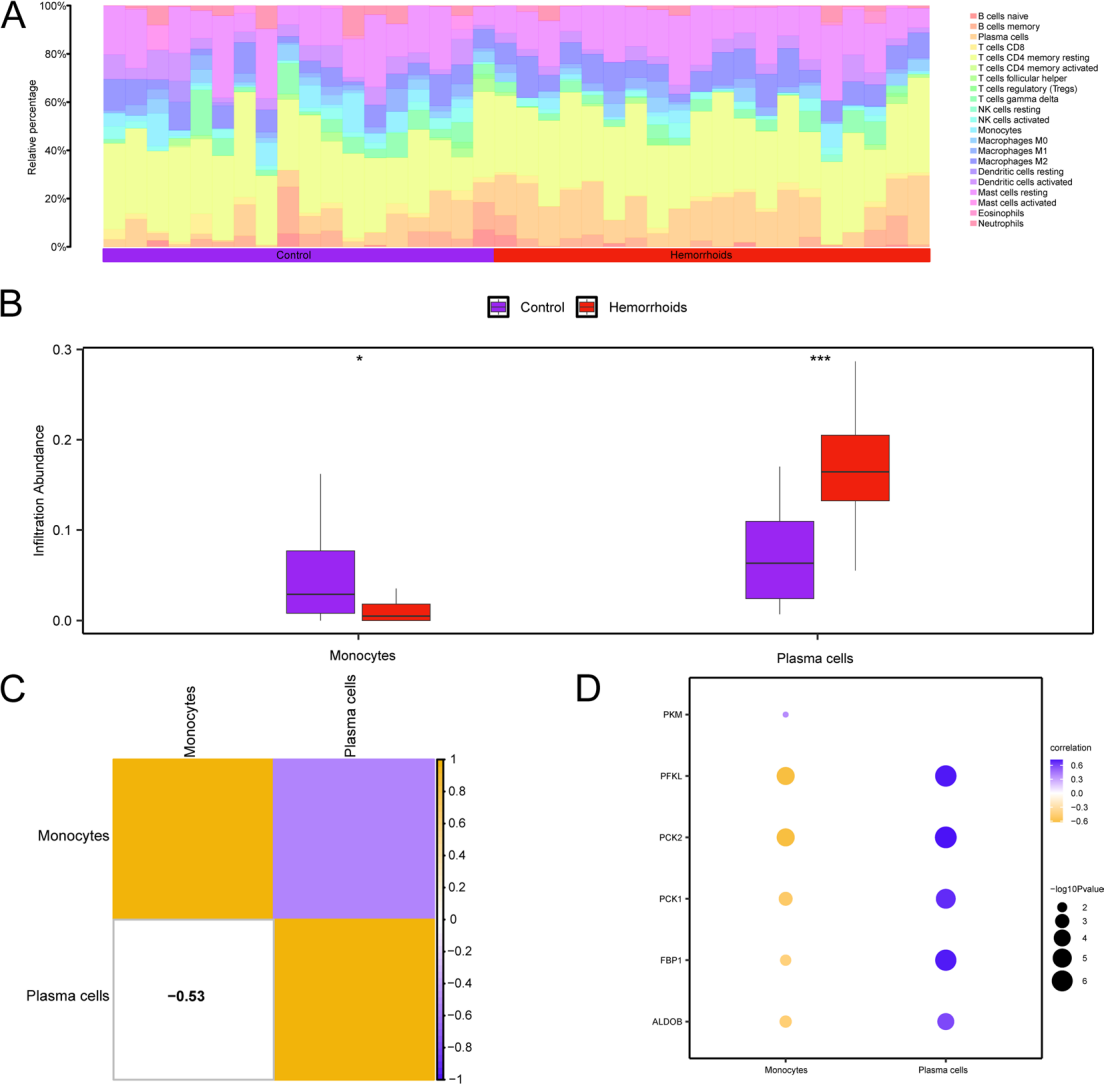
GSE154650 Immune Infiltration Analysis using the CIBERSORT Algorithm. (**A**,**B**) Bar chart (**A**) and group comparison chart (**B**) depict the distribution of immune cell proportions within dataset GSE154650. C. Heat map visualization illustrating the correlation of immune cell infiltration abundance in dataset GSE154650. D. Bubble plot displaying the link between Model Genes and immune cell infiltration abundance in dataset GSE154650. * indicates *p*-value < 0.05, denoting statistical significance; *** signifies *p*-value < 0.001, representing extremely high statistical significance. The absolute magnitude of correlation coefficients (r values) ranging from 0.5 to 0.8 suggests moderate correlation. Purple is the anal fissures (control) sample, and red is the hemorrhoids sample. Blue is a negative correlation, orange-yellow is a positive correlation, and the depth of the color indicates the strength of the correlation.

**Fig. 10:**
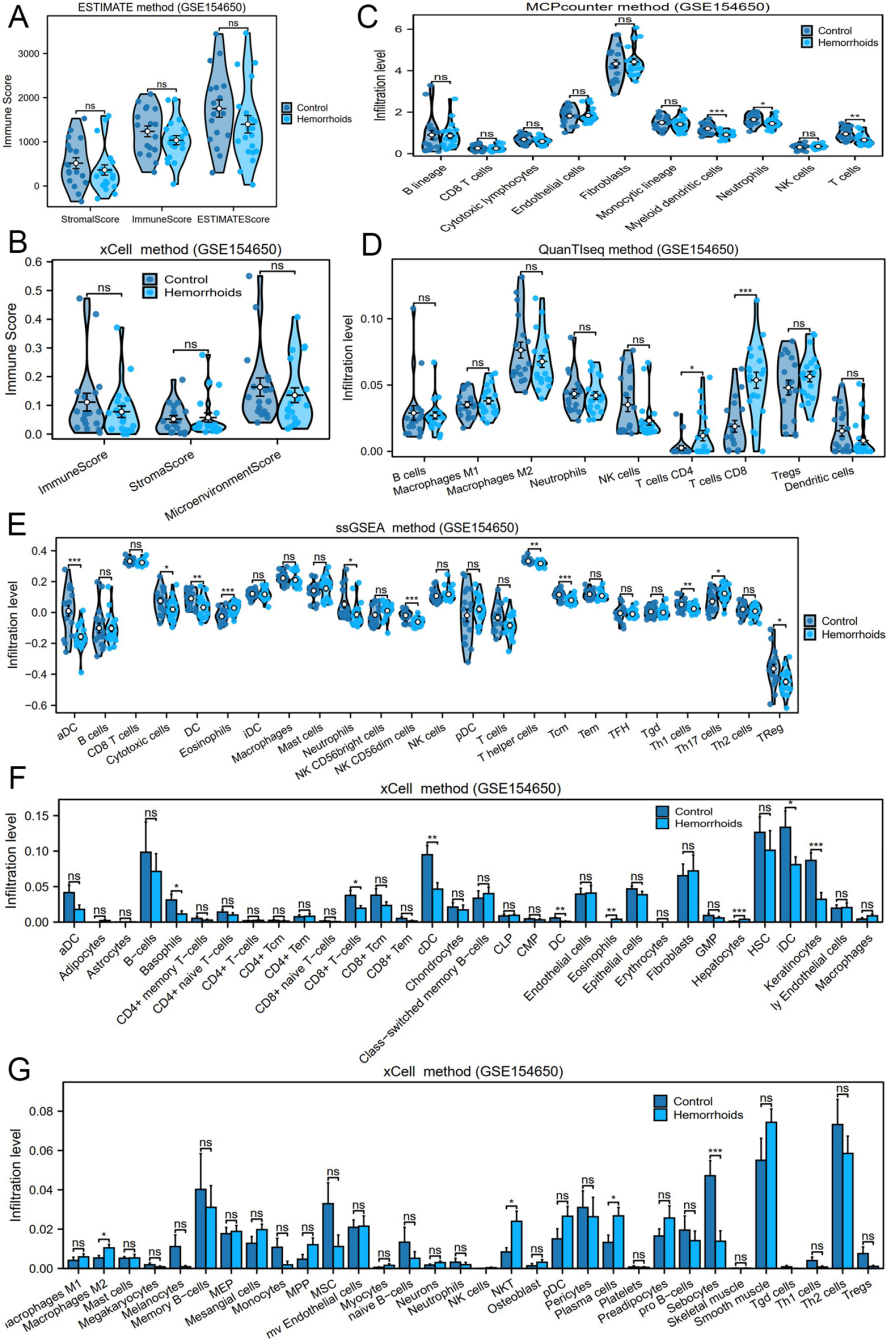
Multi-Method Immune Profiling Reveals Selective Alterations Without Systemic Stromal Changes in Hemorrhoids. Integrated immune infiltration analysis via multiple methods: (**A**) ESTIMATE immune score, (**B**) xCell immune score (both showing no significant differences, *p* > 0.05), (**C**) MCPcounter infiltration level, (**D**) QuanTIseq infiltration level, (**E**) ssGSEA infiltration level, (**F**,**G**) xCell infiltration level; ns = *p* > 0.05, *=*p* < 0.05, **=*p* < 0.01, ***=*p* < 0.001.

The CIBERSORT methodology served to examine the infiltration patterns of 22 specific immune cell populations within the GSE154650 dataset. Following the immune infiltration evaluation, a histogram illustrating the distribution of immune cell proportions within the GSE154650 dataset was constructed (Fig. 9A). The subgroup comparison graph (Fig. 9B) demonstrated the variations in immune cell infiltration abundance between hemorrhoids and control specimens within dataset GSE154650. Analysis revealed marked differences in monocyte level between hemorrhoid and control specimens within dataset GSE154650 (*p* < 0.05). A highly significant disparity was observed for plasma cell levels across groups (*p* < 0.001). Heatmap visualization illustrated the inverse association between these immune cell infiltration patterns (Fig. 9C), with monocytes and plasma cells demonstrating the strongest inverse correlation (*r* = −0.53). Furthermore, bubble plot analysis highlighted HG-immune cell relationships (Fig. 9D): *PFKL* and *PCK2* exhibited a notable positive link to plasma cells (*r* > 0.0, *p* < 0.05), whereas *PFKL* displayed a notable negative link to monocyte abundance (*r* < 0.0, *p* < 0.05). Integrated multi-method analysis (Fig. 10) revealed key patterns: ESTIMATE/xCell showed no significant differences in stromal/immune scores (*p* > 0.05), indicating no systemic microenvironment alterations. However, MCPcounter indicated reduced myeloid dendritic cells, QuanTIseq revealed increased T cells CD8, and xCell detected elevated plasma cells. Discrepancies in specific cell-type alterations across methods suggest selective immune activity changes during hemorrhoid pathogenesis.

## Discussion

Hemorrhoids represent a widespread anorectal disorder with a substantial disease burden in adult populations, characterized by clinical manifestations including pain, hemorrhage, and functional impairment. This underscores the imperative to elucidate molecular drivers and discover novel therapeutic candidates for improved clinical management. Our investigation focused on the interplay between glycolytic processes and hemorrhoidal pathogenesis. Utilizing a systems biology approach, this investigation systematically mapped core glycolytic regulators and associated metabolic networks implicated in disease progression, thereby providing a framework for subsequent mechanistic studies and translational developments aimed at optimizing diagnostic precision and therapeutic outcomes.

Our study delineates *PCK1*, *ALDOB*, and *PCK2* as core regulators, offering critical mechanistic perspectives on hemorrhoid pathogenesis. *PCK1*, a rate-limiting enzyme in gluconeogenesis, catalyzes oxaloacetate-to-phosphoenolpyruvate conversion, a pivotal step in glucose homeostasis. Its overexpression correlates with metabolic perturbations, suggesting involvement in hemorrhoid-associated metabolic reprogramming. Emerging evidence positions *PCK1* as a mediator of inflammatory cascades central to hemorrhoid progression^6^. *ALDOB*, a glycolytic/fructose catabolism regulator, modulates cellular bioenergetics and inflammation via substrate flux adjustments^7^. Its marked overexpression in hemorrhoid tissues aligns with observed glycolytic flux alterations, reinforcing metabolic instability as a hallmark across multifactorial pathologies, including hemorrhoids. *PCK2* is also involved in gluconeogenesis and exhibits significant functions in cellular stress responses; its expression is often increased in response to metabolic stress, suggesting a potential adaptive mechanism in pathological conditions^8^. A thorough investigation into the regulatory mechanisms that control the expression of these genes and their interactions with other metabolic pathways could offer valuable insights for developing targeted therapies aimed at restoring the metabolic balance in patients with hemorrhoids.

KEGG enrichment analysis demonstrated significant associations between the identified GRDEGs and several critical metabolic pathways, notably the glucagon signaling pathway, Glycolysis/Gluconeogenesis, Carbon metabolism, AMPK signaling circuit, and Central carbon metabolism in cancer. The glucagon signaling axis serves as a central modulator of systemic glucose homeostasis and bioenergetic regulation, predominantly through hepatic gluconeogenesis and glycogenolysis coordination. Dysregulation within this axis is associated with metabolic dysfunction, suggesting GRDEGs-mediated perturbations in glucose flux may drive hemorrhoid pathogenesis via energy metabolism alterations^9^. The Glycolysis/Gluconeogenesis pathway operates as a cornerstone of cellular ATP synthesis, particularly during hypoxic stress. Upregulation of glycolytic activators in hemorrhoid tissues likely reflects metabolic reprogramming to sustain exacerbated bioenergetic requirements from inflammatory cascades and stromal restructuring^10^. Furthermore, the AMPK cascade functions as a master regulator of cellular energy sensing, dynamically responding to adenylate charge variations. AMPK activation stimulates energy-yielding catabolism while suppressing biosynthetic anabolism, thereby preserving metabolic flexibility^11^. Notably, central carbon metabolism adaptations mirror mechanisms observed in neoplastic progression, where metabolic rewiring supports pathological expansion. While hemorrhoids lack malignant transformation, these conserved metabolic signatures highlight the universal role of glycolytic adaptation across inflammation-driven pathologies^12^. Elucidating these pathway interdependencies in hemorrhoid biology may enable mechanism-based interventions targeting metabolic vulnerabilities to improve clinical management.

TFs and miRNAs exhibit multifaceted regulatory interplay in modulating GRDEGs expression, notably *PCK1* and *ALDOB*. Core transcriptional controllers of *PCK1* include cAMP response element-binding protein, which orchestrates *PCK1* transcriptional activation via glucagon/cortisol signaling to support gluconeogenesis^13^. *E2F1*, a cell cycle modulator, enhances gluconeogenic gene transcription under metabolic stress^14^while YY1 directly binds the *PCK1* promoter to fine-tune its activity^13^. CCAAT/enhancer-binding protein beta (CEBPB) further amplifies *PCK1* expression during cytokine-driven inflammation, bridging metabolic and immune pathways^15^. These TFs collectively synchronize energy-sensing networks to adapt to fluctuating bioenergetic demands. Conversely, miR-29 suppresses *PCK1* by targeting its 3’ untranslated region (UTR) for mRNA degradation, impairing gluconeogenic capacity—a mechanism implicated in metabolic syndrome pathogenesis where miR-29 overexpression correlates with insulin resistance^16,17^. Emerging evidence highlights miR-29-STAT3 crosstalk as a potential amplifier of TF-mediated *PCK1* regulation, underscoring layered control mechanisms^17^.

TFs exert pivotal control over *ALDOB* expression, with MYC and GATA6 emerging as key players. MYC, a proto-oncogene overactivated in malignancies, drives glycolytic flux amplification while suppressing *ALDOB* transcription—a mechanism implicated in cancer metabolic rewiring toward aerobic glycolysis (Warburg effect)^18^. Conversely, GATA6 enhances *ALDOB* expression, particularly in hepatic tissues, to stabilize metabolic equilibrium^19^. This TF-mediated antagonism (MYC inhibition vs. GATA6 activation) underscores a dynamic regulatory axis influencing both cellular metabolism and oncogenic progression. Such interactions hold clinical relevance in *ALDOB*-deficient malignancies like hepatocellular carcinoma, where *ALDOB* functions as an oncosuppressor^20^. Parallel studies reveal miR-30-mediated *ALDOB* silencing across tumor contexts, linking its downregulation to pro-tumorigenic phenotypes including lipogenesis potentiation and apoptosis resistance^21,22^. Our multi-database intersection identified high-confidence upstream miRNAs, exemplified by hsa-miR-944 targeting MRPS5 (four-database validated; contest + + score = 99). This mitochondrial regulator likely disrupts oxidative phosphorylation in hemorrhoid vasculature, contributing to venous dilation and impaired contractility. Crucially, its suppression of MYC and crosstalk with NF-κB pathways potentially amplify immune cell infiltration—extending PCK1-like regulatory hierarchies to glycolytic-immune axes, with significant implications for hemorrhoid pathogenesis. Expanding investigations to *PCK1*-targeting miRNAs is imperative, given their critical roles in fine-tuning metabolic gene networks through post-transcriptional regulation^23^.

Marked alterations in monocyte and plasma cell infiltration within hemorrhoidal tissues suggest glycolytic pathway modulation of immune cell dynamics in disease pathogenesis. Activated immune cells universally upregulate glycolytic flux to fuel effector functions, sustaining the bioenergetic demands of inflammation and repair^24,25^. In the hemorrhoidal microenvironment, monocytes undergo metabolic adaptation to support pro-inflammatory polarization: enhanced glycolysis facilitates reactive oxygen species (ROS) generation and matrix metalloproteinase-9 (MMP-9) secretion (notably MMP-9 degrades elastin in venous walls), exacerbating vascular leakage and stromal weakening—key features of hemorrhoidal prolapse^26–28^. Similarly, plasma cell infiltration—driven by local B-cell maturation requiring c-MYC/STAT6-mediated glycolytic reprogramming^29,30^—propagates chronic inflammation through autoantibody-induced complement activation and direct fibrogenic signaling: transforming growth factor beta 1 (TGF-β1) from these cells stimulates pathological collagen deposition in submucosa, leading to irreversible tissue stiffening^31^. Critically, this creates a self-amplifying circuit where glycolytic monocytes sustain plasma cell survival via interleukin-6 (IL-6), while plasma cell-derived C-X-C motif chemokine ligand 8 (CXCL8) recruits new monocytes^28,31^. Therapeutic disruption of this immunometabolic crosstalk—targeting monocyte C-C motif chemokine ligand 2 (CCL2) receptors or plasma cell B lymphocyte stimulator (BLyS)/a proliferation-inducing ligand (APRIL) dependence—may resolve the glycolytically-driven inflammation-fibrosis axis that defines recalcitrant hemorrhoid progression. While this self-amplifying circuit exacerbates focal damage, integrated multi-algorithm analyses reveal no diffuse stromal-immune remodeling—confirming pathological confinement to vascular-niche microenvironments. Such targeted alterations suggest immune activity as a disease-amplifying comorbidity rather than primary driver.

The primary constraints of this investigation arise from its dependence on computational predictions without orthogonal experimental verification, precluding definitive validation of the proposed mechanisms. Furthermore, the limited cohort size derived from repository data could constrain statistical robustness, risking spurious correlations. In this context, our selection of a lenient differential expression threshold (|logFC| > 0) aimed to mitigate potential omission of key genes—a strategy particularly valuable for exploratory studies with restricted samples and poorly defined disease pathogenesis. However, such permissive thresholds may elevate false-positive inclusions, introducing noise that necessitates cautious interpretation of downstream analyses. The exclusion of patient-derived specimens restricts the external validity of these findings and their translational relevance to clinical practice. Critically, all reported results require validation under more stringent filtering criteria (e.g., |logFC| > 1, adj.*p* < 0.05) and independent cohorts to establish the biological significance of candidate genes. Subsequent investigations necessitate functional studies to authenticate candidate biomarkers and pathways, thereby elucidating their mechanistic contributions to hemorrhoid pathogenesis. Due to the difficulty in obtaining high-quality datasets, the robustness of the results has been enhanced through methods such as hierarchical cross-validation. If more relevant datasets are made public in the future, external independent validation will be supplemented in a timely manner.

This integrative analysis employed multi-omics approaches to delineate GRGs-hemorrhoid interactions, uncovering critical metabolic axes and central regulatory nodes implicated in disease pathogenesis. These findings lay the groundwork for deeper exploration of the pathways governing hemorrhoid development and may contribute to innovative therapeutic approaches. Future studies should incorporate experimental validations to assess the clinical relevance of the identified targets and their capability to enhance patient outcomes.

## Materials and methods

### Data acquisition

The GEOquery package for R^32^ (Version 2.70.0) from the Gene Expression Omnibus database^33^ (https://www.ncbi.nlm.nih.gov/geo/) was utilized to download the Hemorrhoids, Hemorrhoids GSE154650^34^ sample dataset. The specimens in dataset GSE154650 were from *Homo sapiens*, and all tissues were procured from the Hemorrhoid Tissue. The chip platform for the dataset GSE154650 was GPL20301. For detailed information, refer to Table 1. The dataset GSE154650 contains 20 hemorrhoids and 18 anal fissures (control). All samples were included in the study.

The GeneCards database^35^ (https://www.genecards.org/) and Molecular Signatures Database (MSigDB)^36^ (https://www.gsea-msigdb.org/gsea/msigdb) serves as the primary sources for identifying GRGs. Genes were retrieved from the GeneCards database using “glycolysis” as the search keyword. Filtering criteria were applied to retain only genes designated as “Protein Coding” and with a “Relevance Score > 1”. The Relevance Score quantitatively assesses the association between each gene and the search keyword by integrating evidence from literature, functional annotations, and biological pathways. Setting the threshold > 1 significantly enhances the selection of genes biologically relevant to glycolysis, minimizes the inclusion of low-relevance genes, and thereby increases the scientific rigor and specificity of subsequent enrichment analyses. This established filtering approach has been utilized in previous studies, supporting its validity and applicability. Ultimately, a total of 1,561 GRGs were procured from GeneCards. Similarly, using “glycolysis” as the keyword to search the MSigDB database, we incorporated all relevant authoritative gene sets directly associated with glycolysis, including BIOCARTA_GLYCOLYSIS_PATHWAY, KEGG_GLYCOLYSIS_GLUCONEO GENESIS, REACTOME_GLYCOLYSIS, HALLMMAPK CLYCOLYSUS, REACTOME_REGULATION_OF_GLYCOLYSIS_BY_FRUCTOSE_2_6_BISPHOSPHATE_METABOLISM, WP_AEROBIC_GLYCOLYSIS, WP_AEROBIC_GLYCOLYSIS_AUGMENTED, HALLMARK_GLYCOLYSIS, WP_GLYCOLYSIS_AND_GLUCONEO GENESIS, and WP_GLYCOLYSIS_IN_SENESCENCEWP_HIF1A_AND_PPARG_REGULATION_OF_GLYCOLYSIS. This search yielded an aggregate of 305 GRGs. The GRG sets obtained from both databases were then consolidated, and duplicate entries were removed. A comprehensive analysis led to the identification of 1,711 unique GRGs for subsequent downstream analysis. Comprehensive data is depicted in Supplementary Table S6 online.

### GRDEGs associated with hemorrhoids

Based on the sample categories within the GSE154650 dataset, the samples were classified into two distinct groups: hemorrhoids and anal fissures (control). The R package limma^37^ (version 3.58.1) was applied to assess the differential expression of genes between hemorrhoid and annal fissure samples. Genes exhibiting a logFC above zero combined with adj.*p* values below 0.05 were identified as upregulated, whereas genes showing logFC values below zero alongside adj.*p* values under 0.05 were identified as downregulated^38^. The expression data visualization was executed utilizing the ggplot2 package in R (version 3.4.4).

To acquire GRDEGs associated with hemorrhoids, DEGs exhibiting |logFC| exceeding 0 and an adj. p below 0.05, as determined from the differential analysis of the dataset GSE154650, were subsequently cross-referenced with GRGs, and Venn diagrams were drawn to obtain GRDEGs. A heat map was developed utilizing the pheatmap package for R (version 1.0.12).

### GO and KEGG pathway enrichment analyses

GO analysis^39^ represents a commonly adopted method for conducting extensive functional enrichment investigations, encompassing BP, CC, and MF categories. KEGG^40,41^ functions as an established database that stores data regarding genomes, biological pathways, diseases, and drugs (Copyright permission of KEGG is provided as a related file). The R package clusterProfiler^42^ (Version 4.10.0) was implemented to execute GO and KEGG pathway enrichment analyses of GRDEGs. The selection criteria were adj.*p* < 0.05 and FDR (q value) < 0.25.

### GSEA

GSEA^43^ is a method utilized to examine the allocation patterns of genes from specified gene sets within a gene list arranged according to their association with phenotypic traits. This analytical approach allows the assessment of how much these genes contribute to the observed phenotypes. In this investigation, genes in the dataset GSE154650 were first sequenced according to their logFC values. Afterward, GSEA was conducted for all genes within the GSE154650 dataset employing the clusterProfiler package for R^42^ (version 4.10.0). The GSEA configuration employed these specifications: a seed value of 2022 was utilized, with each gene set comprising a minimum of 10 genes and an upper limit set for a total of 500 genes. Using MSigDB^36^ access to c2.all.v2022.1. Hs.symbols.gmt [Curated/Pathway] (6449) gene set for GSEA, the selection criteria were adj.*p* < 0.05 and FDR value (q value) < 0.25.

### PPI network construction and HG selection

A PPI network consists of proteins that engage with each other through their respective interactions. The STRING^44^ database allows the investigation of interactions between both established and predicted proteins. This investigation utilized the STRING database to establish a PPI network linked to GRDEGs, employing a correlation coefficient threshold greater than 0.7, while establishing the minimal interaction score at low confidence (0.150) as the criterion. In addition, all five algorithms from the cytohubba^45^ plugin of the Cytoscape^46^ software were applied: MCC, Degree, MNC, EPC, and Closeness. In the PPI network analysis, the scores for GRDEGs were initially computed. Afterward, the top 10 GRDEGs were ranked based on these scores and selected for further examination. Finally, the genes identified through the application of the five distinct algorithms were subjected to intersection analysis, and a Venn diagram was constructed for further evaluation. Genes that emerged from the intersection of these algorithms were determined to be pivotal HGs linked to glycolysis.

### Development of regulatory network

TFs control post-transcriptional gene expression through interactions with HGs. We retrieved TFs utilizing the ChIPBase database^47^ (http://rna.sysu.edu.cn/chipbase/) and analyzed their regulatory functions on HGs. Subsequently, the regulatory network involving mRNA and TFs was illustrated utilizing Cytoscape^46^ software.

Furthermore, miRNAs have crucial regulatory functions in biological development and evolutionary processes. These molecules regulate multiple target genes while individual target genes can be controlled by different miRNAs. To examine the link between HGs and miRNAs, HG-related miRNAs were obtained using the TarBase^48^ database (http://www.microrna.gr/tarbase), and the regulatory network involving mRNA and miRNAs was illustrated utilizing Cytoscape software.

Furthermore, to enhance the reliability of upstream miRNA prediction, multiple databases were incorporated beyond TarBase. Specifically, TargetScan^49^ (http://www.targetscan.org), ENCORI^50^ (http://starbase.sysu.edu.cn), and miRDB^51^ (http://mirdb.org) were utilized to predict potential miRNAs regulating HGs. Predictions from each database were retrieved individually, followed by an intersection strategy to identify miRNAs consistently predicted across multiple databases. This approach minimized false-positive predictions and improved robustness. Subsequently, the intersected miRNAs were integrated to construct the mRNA-miRNA regulatory network, while visualization procedures utilizing Cytoscape remained unchanged.

### Expression difference validation of HGs and analysis of ROC curves

To examine the variations in HG expression between hemorrhoids and anal fissure (control) samples in the dataset GSE154650, a comparative grouping map was constructed per the significant HGs level. Finally, the pROC package for R^52^ (version 1.18.5) facilitated the creation of ROC curves for HGs. The ROC curves and AUC values were calculated to assess the diagnostic potential of HG expression in detecting hemorrhoids. Generally, the AUC values of ROC curves range between 0.5 and 1. As AUC values approach 1, they indicate enhanced diagnostic capabilities. AUC values demonstrate restricted accuracy from 0.5 to 0.7, adequate accuracy between 0.7 and 0.9, and superior accuracy when exceeding 0.9. To demonstrate clinical translation potential, confusion matrix analysis was performed to evaluate the 7-HG integrated diagnostic model. Predictions from dataset GSE154650 were used to generate a heatmap quantifying four outcome categories: true positives (correct hemorrhoid identification), true negatives (correct anal fissure identification), false positives (anal fissures misclassified as hemorrhoids), and false negatives (hemorrhoids missed).

### Immunoinfiltration analysis of hemorrhoids (CIBERSORT)

CIBERSORT^53^ utilizes a deconvolution approach applied to a transcriptomic expression matrix grounded in the principles of linear support vector regression. This approach determines the relative proportions and quantities of immune cell subtypes within mixed cell populations. The analysis incorporated the CIBERSORT algorithm alongside the LM22 signature gene matrix for data processing. Subsequently, data points exhibiting an immune cell-enriched fraction exceeding zero were selectively retained. This process yielded specific results pertaining to the immune cell infiltration matrix within the GSE154650 dataset, from which a proportional histogram was generated for visualization purposes. Subsequently, the ggplot2 package for R (version 3.4.4) was applied to generate a comparative visualization, demonstrating the variation in the expression of LM22 immune cell types between hemorrhoids and control specimens, as indicated in the dataset GSE154650. Further examination identified statistically significant distinctions in immune cell populations between hemorrhoids and control cohorts. A Spearman correlation analysis assessed relationships among different immune cell populations, with results displayed through a correlation heatmap generated via the pheatmap package in R (version 1.0.12). The Spearman correlation method examined associations between model genes and immune cell populations, including only results achieving *p*-values below 0.05. The link between model genes and immune cells were visualized via a bubble plot utilizing the ggplot2 package in R (version 3.4.4). To enhance immune infiltration assessment, several supplementary methods were applied: ESTIMATE, MCPcounter, QuanTIseq, ssGSEA, and xCell. All analyses leveraged the GSE154650 dataset.

### Statistical analysis

All data processing and analyses presented in this paper were conducted using the R software (version 4.2.2). Unless otherwise specified, the statistical significance of normally distributed continuous variables was evaluated using the independent Student’s t-test. In contrast, the Mann-Whitney U test also referred to as the Wilcoxon rank-sum test, was applied to examine differences in non-normally distributed variables. Comparisons among multiple groups were conducted using the Kruskal-Wallis test. Correlation coefficients between different molecules were determined through Spearman’s correlation analysis. Statistical *p*-values were bilateral unless specified differently, with *p* < 0.05 indicating statistical significance.

## Supplementary Information

Below is the link to the electronic supplementary material.

Supplementary Material 1

Supplementary Material 2

## Data Availability

All data produced and examined throughout this investigation is accessible within the published manuscript and accompanying supplementary documentation (Supplementary Tables S1–S6 and AUC confidence interval in ROC analysis are provided as supplementary files).
