# Supplementary material for: Role of glycolysis related genes in the pathogenesis of hemorrhoids and immune cell infiltration analysis
Source: Sci Rep. 2025 Sep 25;15:32912. doi: 10.1038/s41598-025-18382-3 (PMC12464167; doi:10.1038/s41598-025-18382-3)
Supplement: Supplementary file 1 — Supplementary Material 1 [file 41598_2025_18382_MOESM1_ESM.docx]

| Hub Gene | AUC | CI |
| --- | --- | --- |
| PCK1 | 0.928 | 0.851 – 1.000 |
| ALDOB | 0.814 | 0.676 – 0.952 |
| PCK2 | 0.811 | 0.671 – 0.951 |
| PFKL | 0.831 | 0.691 – 0.970 |
| PKM | 0.772 | 0.612 – 0.932 |
| ENO1 | 0.742 | 0.581 – 0.902 |
| FBP1 | 0.769 | 0.616 – 0.923 |

AUC confidence interval in ROC analysis

When the AUC is greater than 0.5, it indicates that the expression of the molecule is a tendency to promote the occurrence of events. The closer the AUC is to 1, the better the diagnostic effect. When the AUC is between 0.7 and 0.9, it has a certain degree of accuracy; when the AUC is above 0.9, it has relatively high accuracy. AUC, Area Under the Curve; CI, Confidence Interval.
